# Sexual and gender minority cultural humility training for oncology settings: An example of iterative adaptation and implementation

**DOI:** 10.3389/frhs.2022.958274

**Published:** 2022-11-10

**Authors:** Charles S. Kamen, Melhaney Reichelt, Porooshat Dadgostar, Ash B. Alpert, Christopher Doucette, Phillip Vaughan, Alex S. Keuroghlian, Reza Yousefi-Nooraie

**Affiliations:** ^1^University of Rochester, Rochester, NY, United States; ^2^Brown University School of Public Health, Providence, RI, United States; ^3^The Fenway Institute, Boston, MA, United States

**Keywords:** cancer, sexual orientation, gender identity, health disparities, sexual and gender minorities, cultural humility

## Abstract

**Background:**

Multiple national organizations recommend that cancer care providers and oncology practices be responsive to the needs of sexual and gender minority (SGM) patients. Oncology practices have attempted to incorporate this recommendation through SGM-focused cultural humility training interventions. It is unclear how best to adapt and implement such training across practices. This manuscript outlines one process for adapting a widely-used SGM training from The Fenway Institute to the context of oncology settings using the Framework for Reporting Adaptations and Modifications-Enhanced (FRAME) model.

**Methods:**

We conducted training sessions in two oncology care settings: a breast oncology center and a radiation oncology department. Subsequently, we conducted in-depth interviews with the three trainers involved in adapting The Fenway Institute's training to these two practices. Two independent investigators coded the interviews using components of the FRAME model as an analytic guide.

**Results:**

Training team members described the mechanisms by which FRAME adaption occurred both proactively and reactively; the importance of involving SGM-identified trainers of diverse backgrounds as well as champions from within oncology practices in which trainings were conducted; the importance of adapting both the context and content of training to be relevant to oncology audiences; and the ways in which fidelity to the core principles of improving health care for SGM patients was maintained throughout the process.

**Discussion:**

SGM cultural humility training for oncology providers and staff must undergo iterative adaptation to address the political and social context of specific practice environments and advocate for broader institutional culture change to achieve responsiveness to SGM health needs.

## Introduction

Sexual and gender minority individuals (SGM; e.g., lesbian, gay, bisexual, transgender, queer; LGBTQ+) experience high rates of psychological distress, low rates of insurance coverage, and difficulty accessing culturally competent and culturally humble healthcare services (1–6). These same disparities affect SGM people with cancer, reducing access to oncology care, quality of life following cancer care, and, potentially, rates of survival from cancer (7–9). Some studies have found that SGM cancer patients report higher psychological distress, depression, and anxiety than heterosexual and cisgender patients (i.e., those who are primarily attracted to people of genders different from their own and whose gender identities match societal expectations based on their sex assigned at birth; H/C) (10, 11). This is a major concern given the link between higher psychological distress and increased risk of mortality from cancer (12–14). These studies also highlight unique factors that affect distress for SGM cancer patients (15–17). One unique factor is minority stress, or chronic stress arising from experiences of prejudice and discrimination based on sexual orientation or gender identity (2, 5). Pre-existing disparities in distress, caused by minority stress, may be exacerbated by stigma and discrimination experienced during cancer diagnosis and treatment (e.g., discrimination from cancer care providers based on sexual orientation and/or gender identity) (18–21). In the face of minority stress, SGM cancer patients have asked for providers to “treat us with dignity” (22).

Given this request, interventions to improve SGM cultural competency and humility of oncology personnel as they treat SGM patients are urgently needed. Throughout this manuscript we will refer to “cultural humility” as the preferred approach to training interventions. Cultural humility training emphasizes awareness of trainees' personal biases, patient-centeredness, and openness to lifelong learning (23). The literature on racial/ethnic minority cultural humility training interventions highlights that such interventions are effective in improving provider knowledge and skills (24, 25) as well as patient satisfaction with care (26, 27). Importantly, satisfaction with care is a fundamental component of high-quality care, underscoring the importance of promoting cultural humility training (28, 29). While the literature on SGM humility training is still in its infancy (24, 30), based on limited evidence, SGM humility training has been shown to be effective in improving clinicians' knowledge and attitudes regarding SGM patients (31–35). Such training must also acknowledge that SGM identities also cut across all populations and that SGM people with multiple marginalized identities experience multiplicative marginalization and barriers to care (36). Examples of populations with intersecting marginalized identities include SGM people of color (37), SGM people who are economically disadvantaged (38, 39), or SGM people with disabilities (40). To date, no studies have tailored intersectional SGM cultural humility training specifically to the context of oncology (41).

Despite limited data on the efficacy of training, cancer care facilities have begun to mandate SGM cultural humility training in response to the requests of patients and clinicians (42). The Fenway Institute's (TFI) National LGBTQIA+ Health Education Center has been at the forefront of delivering SGM-relevant training to healthcare facilities nationwide (43–45). Their SGM humility training intervention is based on a decade of program evaluation in non-cancer healthcare settings and was developed with a diverse community advisory board, based on survey data, chart review, and literature reviews (44, 46, 47). TFI's intervention focuses on four components, which are presented in Table 1. The TFI intervention has not included oncology-specific examples and has not been evaluated in the context of oncology.

**Table 1:** Core components of SGM cultural competence training.

**SGM training core components**	**TFI's original curriculum**
SGM concepts and terminology	Didactic presentation, printed glossary
Health disparities among SGM patients	Didactic presentation of population-based data
How to use sexual orientation and gender identity (SOGI) data in clinical practice	SOGI data collection toolkit, SOGI data collection demonstration videos, SOGI case studies
Improving the environment for SGM patients	Non-discrimination policy language, SGM patient experience video

To address this gap, we adapted TFI modules to address specific issues confronted by diverse SGM patients in oncology settings. In this article, we report on the process of iterative adaptation and implementation of TFI's SGM cultural humility training modules in two different oncology contexts: a breast oncology center and a radiation oncology department. We use the Framework for Reporting Adaptations and Modifications-Enhanced (FRAME) as a foundation for documenting and reporting our adaptation efforts (48). We present the results of our adaptation in order to establish a roadmap that other groups can follow when adapting TFI or other cultural humility training interventions to their specific healthcare contexts.

## Methods

### Initial adaptation of intervention

A team of four clinicians and scientists (AA, a Non-Hispanic White, queer, non-binary person who is a medical oncologist; CD, a Non-Hispanic White, gay cisgender man who is a radiation oncologist; CK, a Non-Hispanic White, gay cisgender man who is a clinical psychologist; and PV, a Non-Hispanic Black, gay cisgender man who was a public health graduate student) came together to implement a series of SGM-focused cultural humility trainings for oncology practices in the Wilmot Cancer Institute care network. The core training materials had been developed by TFI as described above. Oncology-specific content included in the training was based on feedback from a mixed-methods study conducted by the National LGBT Cancer Network (22, 49), findings from qualitative interviews with SGM cancer patients, their caregivers, and their providers (50), a focus group of transgender and gender diverse individuals affected by cancer (51), and the clinical experiences of the team members. The team also discussed pragmatic aspects of adaptation to address the needs of different oncology clinics. The training was implemented at two regional care locations in the Wilmot Cancer Institute (Wilmot) network in upstate New York.

### Ethical review

These trainings and subsequent data collection were approved by the Institutional Review Board of the University of Rochester.

### First training

Three training team members (CD, CK, PV) delivered a 1-h training session to breast oncology clinicians and staff. Sixteen personnel attended, including six nurses, four social workers, four administrative staff, and two medical oncologists. The delivery and presentation of training materials took place in a conference-style room with a single large table for attendees and trainers, as well as a wall-mounted screen for the slide presentation.

### Second adaptation

Four training team members (AA, CD, CK, PV) reviewed the findings from the first training. Based on personal reflections of the trainers and comments from attendees, the training materials were further adapted before the second training session.

### Second training

Two training team members (CD, CK), delivered a 1-h training session to a radiation oncology department. Forty-one personnel attended, including 14 nurses, 12 radiation oncologists, eight administrative staff, four administrators, and three dosimetrists. The delivery and presentation of training materials took place in a lecture-style classroom with tables for attendee seating, a projection screen, and a podium for the trainer(s) speaking.

### Follow-up interviews

After the second training, two authors (MR, PD) conducted in-depth semi-structured interviews with three training team members (CD, CK, PV), all of whom are also authors on this paper. The interview guide was based on the FRAME model for adapting interventions and the purpose of the interviews was to capture the team's reflections on the adaptation process. Interviews were audio-recorded using Zoom and transcribed using otter.ai software, along with coder review. We analyzed transcripts in Dedoose, a qualitative data analysis program, using the components of FRAME as an explicit guide for analysis (48, 52). Two coders (MR, PD) independently reviewed the three interviews using Dedoose to extract quotes that exemplified the different components of the FRAME model. The coders discussed and refined these quotes collaboratively to create a preliminary codebook consisting of 36 codes, which both coders consistently applied to the 3 interviews. Two auditors (CK, RYN) reviewed the data to assess whether codes aligned with the quotes from interviewees; based on this feedback, final codes were aligned with the components of the FRAME model, and a table was generated based on the modular structure of the FRAME-Implementation Strategies tool (FRAME-IS) (53). The data presented here include the final set of FRAME components, relevant codes, and illustrative quotes, which have been minimally edited for readability.

## Results

We structure the results based on the domains of the FRAME model and tabulate the results based on FRAME-IS modules. The table is comprised of 7 different modules used to document modifications to implementation strategies: a brief description of the EBP, implementation strategy, modification, and the reason for modification (Module 1); what is modified (Module 2); the nature of modification and the relationship between modifications and core-implementation strategies (Module 3); the goals and rationale for modification (Module 4) when the modification occurred, and whether it was planned (Module 5); who participated in the decision to modify (Module 6); and how widespread the modification is (Module 7). We present the modules in Table 2 using the order outlined in the original FRAME-IS manuscript, but for readability, we present the results below in the order in which topics were discussed by the interviewees. Interviewees offered further insights that were not captured by the existing FRAME model; these comments are presented as a separate section in the text and FRAME-IS table.

**Table 2:** Adaptation of SGM cultural humility training according to FRAME-IS.

**Module 1:**		**Specific examples**
• The EBP being implemented is:	The Fenway Institute “Foundations of LGBTQIA+ Health” Training Program	“We didn't use [the TFI training video]…we asked a rhetorical question, ‘does it matter if someone who has cancer is LGBT?”
• The modification(s) being made are:	Tailoring of training content, duration, and environment	Training shortened to 1-h lunch block, one time: “We made it short. We made it over lunch. And those are both implementation
• The reason(s) for the modification(s) are:	To fit the training to oncology audience To fit the training to the schedules of providers and motivate attendance	modifications to try to ensure that people would actually come to the training.”
**Module 2:**		**Specific examples**
• What is being modified?	Content and context of the TFI training	“We wanted to make sure we included issues that would be directly relatable for types of medical diagnoses and issues that they would be dealing with during their care…like talking about sexual side effects of radiation and how that could affect patients and their caregivers.”
**Module 3:**		**Specific examples**
• What is the nature of the content, evaluation, or training modification?	Content modified to include oncology-specific examples Context modified to be shorter, including food, and focused on diverse oncology staff members Reactive adaptation to focus on SGM resilience	Setting changed to be “more of a seminar style than a lecture style.” Provided food for attendees so “they were learning while they were eating, taking care of two things at once.” Multiple people lead the training sessions “that way one person could lead the training two other people could gauge the room, assess, provide feedback, change the slide deck, and when the next person is leading the other two could switch off” “Tried to make some of the examples more positive, as opposed to being gloom and doom”
• What is the relationship to core elements?	Fidelity maintained; adapted intervention consistent with core intervention goals	(See Table 1)
**Module 4:**		**Specific examples**
• What are the goals?	Improve feasibility Improve fit with recipients Increase engagement and satisfaction	“We were specifically training the providers to be able to address issues during their individual [oncology] appointments with patients.”
• What is the level of the rationale for the modification?	Modification based on perceived provider and patient needs	
**Module 5:**		**Specific examples**
• How many times did modification occur?	Twice	Proactive modification of TFI before 1st workshop: “we had a total of three meetings over the course of a month”
• When was the modification initiated?	Planned proactive (pre-implementation/planning) and unplanned reactive (during implementation)	Modifications revisited in 1 meeting between workshops: “we met to discuss our opinions and feedback”
• Was the modification planned?		
**Module 6:**		**Specific examples**
• Who participates in the decision to modify?	Training team members, including practice champion	“We were all members of the LGBTQ community and who were knowledgeable in LGBTQ cultural competency”
• Who makes the ultimate decision?	Same as above	
**Module 7:**		**Specific examples**
• How widespread is the modification?	Based on two separate practices; modified for providers/staff who would have patient contact	“We hoped to reach everyone at the practice from the front desk staff to the chair of department”

### When did adaptation occur?

Team members stated that adaptation of TFI's SGM cultural humility training occurred both proactively and reactively. To proactively adapt the training, the team met in person at the beginning of 2020 for a total of 3 meetings over the course of a month. The modifications focused primarily on program materials, which were adapted before their implementation to make their content relevant to oncology clinicians and staff. Visuals such as pictures and cartoons from the TFI materials were adapted to include cancer patients and caregivers of diverse racial and ethnic backgrounds in order to highlight intersectionality and make SGM people of color visible. Following the first training, several reactive modifications were made in response to feedback from the training team and attendees at the first training.

### Who was involved in adaptation?

The team adapting the training was composed of faculty members and one graduate student from the University of Rochester Medical Center. As described above, all were members of SGM communities. Their training backgrounds included medical oncology, radiation oncology, clinical psychology, and public health. One member of the team (CK) was “the primary person leading the changes in the modifications. He had previously done similar training across the country in [LGBTQ+] cultural competency and wanted to tailor the program to the staff that we would be training…at the University of Rochester Medical Center” (CD). However, the team viewed the experience of adaptation as “pretty collaborative,” because “we were all intended to participate in providing the training,” and “having multiple people thinking about the tailoring ended up making it much stronger” (PV). Team members described the ways their diverse disciplinary viewpoints, alongside their shared lived experience as SGM people, informed their adaptation of the training: “All four of us were very knowledgeable in the area of LGBT cultural humility, and all four of us are also members of the LGBTQ community, so we knew what things we would want to see in a training as both healthcare providers and members of the community” (PV). In preparing for the second training, a team member (CD), who was a resident in the Department of Radiation Oncology, took a lead role in suggesting new examples relevant to radiation therapy, “bringing a focus on actual provider interest in behavior–like what does an oncologist need to know about sexual side effects for LGBT people after radiation” (CK). Team members reflected that including a champion from within the clinic being trained enhanced the success of this second training: “I just have a very supportive department but I'm also engaged with them…I'm telling people, and then it was really just like, word of mouth” (CD).

### What was adapted and what was the nature of the adaptation?

Both the format and the content of the training were adapted to work within oncology settings. In terms of format, the team distilled the 2-h TFI training into a 1-h session. This decision was based on feedback collected before the training from staff and providers at the clinics that they needed the training to be shorter: “People were like, ‘We [staff and providers] cannot take a ton of time away from the clinic, please do it in a short burst, over lunch. We can make that work with the clinic schedule”' (CK). The training team also provided food to participants, an aspect added to encourage the attendance of providers and staff with very busy schedules: “We [the training team] offer Panera sandwiches, so I think that galvanized some people to come who wouldn't have come otherwise” (CK).

In terms of content, the training team iteratively adapted training materials (e.g., PowerPoint slides, handouts) to include content specific to the needs of different oncology audiences. After the first training at the breast oncology center, the training team mutually felt that the examples they had been using were too “negative” and “gloomy.” Therefore, the trainers decided to change the SGM-specific cancer examples to highlight resilience among SGM patients: “We need to revise the content enough so that it … doesn't frighten people away from thinking about [gender and sexuality] issues” (PV). Before the second training, they also changed the content to be specific to radiation oncology. As the participants at this second training were radiation oncologists, dosimetrists, staff, nurses, and a department leader, the trainers added content about the sexual side effects of radiation and ways in which these side effects could uniquely impact SGM patients and their caregivers.

### What were the reasons for the adaptation?

Trainers explained that the primary reason behind the modifications of the TFI model was to increase applicability for the audience: “The Director of Education at Fenway…and I had a long conversation about how Fenway does their training. And he himself said, ‘You know, really these trainings are most effective when they are adapted to the specific healthcare audience where you're trying to deliver them”' (CK). Other reasons for adapting the training included improving feasibility (e.g., length of training), improving perceived applicability to recipients (e.g., oncology examples), addressing sociocultural differences between practices (e.g., all cisgender women at the breast oncology center training), and acknowledging the diversity of SGM cancer patients' experiences, with attention paid to intersectionality. In order to highlight the diversity of patient experiences, the training selected examples derived from interviews with actual SGM cancer patients (50): “We really tried to make it relevant to the trainees that you should care about your LGBTQ patients because look at these things that can happen when you don't, including some obvious discrimination that had occurred” (CD).

### How was intervention fidelity ensured?

The core components of the TFI training program (Table 1) were maintained for this training. In both the original and our adapted version of the training, content addressed SGM terminology, SGM cancer disparities, sexual orientation and gender identity (SOGI) data collection, and institutional non-discrimination policies on the basis of SOGI. Data from the trainings were shared back with leaders at TFI to confirm fidelity with their approach: “He [the TFI Director of Education] had not up to that point done any modification to be oncology specific… it became pretty clear that should be done and we thought, ‘who better to do it than our modification team?”' (CK).

### What were the results of the adaptation?

The training team felt that the adaptation increased participant engagement and improved knowledge gain. Team members reported receiving “verbal feedback” that attendees were “really appreciative of the information that we provided,” that they “actually seemed to gain some confidence in the knowledge portion,” and “they felt much more comfortable with being able to better serve the LGBTQ patient population” (CD). Pre- and post-training surveys (not published) showed significantly higher scores of trainee knowledge and self-efficacy, and reported satisfaction was high (average satisfaction score of 95% out of 100%).

### Reflections on the next iteration

In addition to describing various aspects of the adaptation, interviewees also commented on other ways they would like to augment the training in future. Interestingly, many of these suggestions are directed at improving uptake and implementation, which are not explicitly addressed by FRAME. All three interviewees suggested follow-up training to reinforce skills, saying for example: “In the future, we can make the training more of a series…in the sense that we do one training on one day and then schedule a follow up [training] maybe two months later, or three months later” (PV). Trainers believed these future trainings could be more specific in scope than the initial training, e.g., could focus only on sexual orientation and gender identity data collection or on SGM relationships. They also suggested “doing more…small group… activities” rather than relying primarily on didactic lectures (CK). Interviewees also commented on the need for better training evaluation processes to inform future adaptations: “One thing that we could do…in the future is to have the…posttest as soon as possible [after the training], possibly on REDCap or some other electronic platform” (PV). Finally, interviewees commented that the early inclusion of champions from each practice receiving the intervention would assist with increasing buy-in from administrators and staff and could improve attendance. Additionally, this would increase the applicability of the trainings.

“One of the things that was so successful with the radiation oncology training was having [CD], a fellow in Radiation Oncology, be one of the trainers and promote the training within his facility. …From an implementation perspective, having buy-in from someone inside, preferably somebody with…clout or leadership, makes a huge difference. And, I'd like to think about capitalizing on that, and going up a level to the leadership of the clinic and having the champion connect me to that leader, so that we can, ideally, get the training to be made, if not mandatory, at least strongly encouraged for everybody to attend” (CK).

## Discussion

In this manuscript, we describe an iterative process of adapting and implementing a SGM humility training in two different oncology settings. Our hope is to provide a roadmap that other trainers and implementation scientists can follow when adapting such training programs for their own settings. We used the FRAME model to structure interviews with team members involved in adaptation and implementation, as well as to organize qualitative findings. Commentary from the training team also expanded beyond the FRAME model to cover the importance of iterative adaptation, reflection, and future directions.

As healthcare systems expand regionally, incorporating multiple practices across a large geographic area, efforts to implement interventions may need to account for iterative adaptation on a practice-by-practice basis. Emerging models like FRAME help organize and document the process of such iterative adaptations. Process models also facilitate communication about and generalization of adaptation to other contexts. Given the different contexts of these practices, models like FRAME should be re-applied and the trainings revised to incorporate new lessons learned after each implementation. This is particularly true for interventions addressing diversity, equity, and inclusion, which may need to consider practices' different geographical, political, and social factors. For example, in the current study, we adapted our SGM humility intervention first for a multidisciplinary breast oncology practice, including attendees from the fields of nursing, social work, and oncology, with many cisgender women on staff serving primarily cisgender women; discussing issues like breast cancer in transgender men was relevant here. Second, we adapted for a radiation oncology practice, including radiation techs and front desk staff, with a large proportion of cisgender men on staff serving a more diverse patient base; talking about a range of sexual side effects of treatment was relevant here.

In this exercise, we found it difficult to separate adaptation and implementation, given the dynamic relationships between these processes. Reflecting on the use of FRAME as a qualitative analysis tool, we believe that this conceptual model could be enhanced by incorporating longitudinality and integrating adaptation with implementation. For example, as an intervention is implemented, it should be evaluated for potential adaptation to other care contexts. Future efforts to adapt and implement cultural humility training, specifically, should attend to the interplay between adaptation (e.g., accounting for the practice-level political and social factors mentioned earlier) and implementation (e.g., reach, effectiveness/outcomes).

One important goal for adapting the TFI SGM cultural humility training program for oncology settings was to maximize and facilitate implementation. Interviewees identified several factors that would aid in the implementation of future SGM oncology training programs for clinicians and staff, and these were incorporated into adaptation. For example, providing evaluation results in the form of post-training feedback to participants, as well as engaging department leaders and internal champions, are well-recognized implementation strategies that were suggested as adaptation activities (54). In our adaptation, including an internal champion from one practice allowed the training team to adapt the content of the intervention further to the needs of the practice, facilitating uptake and adoption as measured by verbal feedback about the relevance of the material. This point further highlights the interconnectedness of adaptation and implementation. Both are critical, intertwined, and mutually reinforcing.

### Limitations

The present manuscript presents one example of this adaptation and implementation process. Results are based upon interviews with three team members who conducted two training sessions within a single regional cancer network. Interviews do not allow for collection of observational data and we did not assess the impact of trainings on trainees' behavior. Thus, the lessons learned from the adaptation and implementation of these training sessions may vary in their relevance to other cancer care settings. Finally, the end goal of cultural humility training is to improve patients' experiences with care, and so future research should incorporate the perspectives of patients about their relationship with trained, culturally humble providers.

## Conclusion

The current study provides a real-world example of the adaptation and implementation of an SGM cultural humility training intervention in oncology. Adaptation of this sort of intervention is affected by issues such as the political climate of practices, biases of attendees, and the ongoing societal stigma that surrounds the assessment of sexual orientation and gender identity. Our adaptation methodologies balanced the needs of a cancer care audience with the goal of remaining faithful to the widely-used TFI cultural humility training intervention's core principles. Such training interventions, however, are only one aspect of the systemic and structural reform needed to ameliorate cancer-related disparities affecting SGM populations. These training interventions must coincide with culture change in cancer care practices for all members of the oncology team, including practice leadership, clinicians, front-line staff, and support staff. Practices must create an environment that not only accepts diversity based on sexual orientation and gender identity, but celebrates it. Cultural humility training programs must look beyond practice-level change to ascertain the impact of training on SGM patients' cancer outcomes. Future adaptations of SGM cultural humility training interventions for oncology must aim to incorporate these endpoints if we hope to achieve true health equity for SGM cancer patients.

## Data availability statement

The raw data supporting the conclusions of this article will be made available by the authors, without undue reservation.

## Ethics statement

The studies involving human participants were reviewed and approved by University of Rochester Research Subjects Review Board. The participants provided their written informed consent to participate in this study.

## Author contributions

Data collection and analysis were performed by MR and PD, with supervision by RY-N. The first draft of the manuscript was written by CK. All authors contributed to the study conception and design, commented on previous versions of the manuscript, and read and approved the final manuscript.

## Funding

This research was supported by National Cancer Institute grants K07 CA190529, UG1 CA189961, and UG3 CA262602.

## Conflict of interest

The authors declare that the research was conducted in the absence of any commercial or financial relationships that could be construed as a potential conflict of interest.

## Publisher's note

All claims expressed in this article are solely those of the authors and do not necessarily represent those of their affiliated organizations, or those of the publisher, the editors and the reviewers. Any product that may be evaluated in this article, or claim that may be made by its manufacturer, is not guaranteed or endorsed by the publisher.

## References

[1] Meyer IH. Minority stress and mental health in gay men. J Health Soc Behav. (1995) 36:38–56. 10.2307/2137286 7738327

[2] Meyer IH. Prejudice, social stress, and mental health in lesbian, gay, and bisexual populations: conceptual issues and research evidence. Psychol Bull. (2003) 129:674–97. 10.1037/0033-2909.129.5.674 12956539 PMC2072932

[3] Kertzner RM Meyer IH Frost DM Stirratt MJ. Social and psychological well-being in lesbians, gay men, and bisexuals: the effects of race, gender, age, and sexual identity. Am J Orthopsychiatry. (2009) 79:500–10. 10.1037/a0016848 20099941 PMC2853758

[4] Meyer IH Dietrich J Schwartz S. Lifetime prevalence of mental disorders and suicide attempts in diverse lesbian, gay, and bisexual populations. Am J Public Health. (2008) 98:1004–6. 10.2105/AJPH.2006.096826 17901444 PMC2377299

[5] Hatzenbuehler ML. How does sexual minority stigma “get under the skin”? A psychological mediation framework. Psychol Bull. (2009) 135:707–30. 10.1037/a0016441 19702379 PMC2789474

[6] Gonzales G Henning-Smith C. The affordable care act and health insurance coverage for lesbian, gay, and bisexual adults: analysis of the behavioral risk factor surveillance system. LGBT Health. (2017) 4:62–7. 10.1089/lgbt.2016.0023 27996379

[7] Brown C Mayer DK. Are we doing enough to address the cancer care needs of the LGBT community? Clin J Oncol Nurs. (2015) 19:242–3. 10.1188/15.CJON.242-243 26000571

[8] Boehmer U Elk R. LGBT populations and cancer: is it an ignored epidemic? LGBT Health. (2015) 3:1–2. 10.1089/lgbt.2015.0137 26698385

[9] Alpert AB Scout NFN Schabath MB Adams S Obedin-Maliver J Safer JD. Gender- and sexual orientation- based inequities: promoting inclusion, visibility, and data accuracy in oncology. Am Soc Clin Oncol Educ Book. (2022) 42:1–17. 10.1200/EDBK_350175 35658501

[10] Kamen CS Peoples AR Tejani MA Flannery M Janelsins MC Peppone LJ. Disparities in psychological distress among lesbian, gay, bisexual and transgender (LGBT) cancer survivors. Ann Behav Med. (2014) 47:S245. 10.1002/pon.3746 25630987 PMC4517981

[11] Ussher JM Perz J Kellett A Chambers S Latini D Davis ID. Health-related quality of life, psychological distress, and sexual changes following prostate cancer: a comparison of gay and bisexual men with heterosexual men. J Sex Med. (2016) 13:425–34. 10.1016/j.jsxm.2015.12.026 26853048

[12] Barrera I Spiegel D. Review of psychotherapeutic interventions on depression in cancer patients and their impact on disease progression. Int Rev Psychiatry. (2014) 26:31–43. 10.3109/09540261.2013.864259 24716499

[13] Hamer M Chida Y Molloy GJ. Psychological distress and cancer mortality. J Psychosomat Res. (2009) 66:255–8. 10.1016/j.jpsychores.2008.11.002 19232239

[14] Chida Y Hamer M Wardle J Steptoe A. Do stress-related psychosocial factors contribute to cancer incidence and survival? Nat Clin Pract Oncol. (2008) 5:466–75. 10.1038/ncponc1134 18493231

[15] Kamen C Jabson JM Mustian KM Boehmer U. Minority stress, psychosocial resources, and psychological distress among sexual minority breast cancer survivors. Health Psychol. (2017) 36:529–37. 10.1037/hea0000465 28165265 PMC5444950

[16] Boehmer U Glickman M Milton J Winter M. Health-related quality of life in breast cancer survivors of different sexual orientations. Qual Life Res. (2012) 21:225–36. 10.1007/s11136-011-9947-y 21660650

[17] Matthews AK Hotton A Li CC Miller K Johnson A Jones KW. An internet-based study examining the factors associated with the physical and mental health quality of life of LGBT cancer survivors. LGBT Health. (2016) 3:65–73. 10.1089/lgbt.2014.0075 26789396

[18] Hann D Winter K Jacobsen P. Measurement of depressive symptoms in cancer patients: evaluation of the Center for Epidemiological Studies Depression Scale (CES-D). J Psychosomat Res. (1999) 46:437–43. 10.1016/S0022-3999(99)00004-5 10404478

[19] Patterson JG Jabson Tree JM Kamen C. Cultural competency and microaggressions in the provision of care to LGBT patients in rural and appalachian Tennessee. Pat Educ Counsel. (2019) 102:2081–90. 10.1016/j.pec.2019.06.003 31208771

[20] Lick DJ Durso LE Johnson KL. Minority stress and physical health among sexual minorities. Perspect Psychol Sci. (2013) 8:521–48. 10.1177/1745691613497965 26173210

[21] Katz A. Gay and lesbian patients with cancer. Oncol Nurs Forum. (2009) 36:203–7. 10.1188/09.ONF.203-207 19273409

[22] Kamen CS Alpert A Margolies L Griggs JJ Darbes L Smith-Stoner M. “Treat us with dignity”: a qualitative study of the experiences and recommendations of lesbian, gay, bisexual, transgender, and queer (LGBTQ) patients with cancer. Support Care Cancer. (2019) 27:2525–32. 10.1007/s00520-018-4535-0 30411237 PMC6506401

[23] Murray-Garcia J Tervalon M. The concept of cultural humility. Health Affairs. (2014) 33:1303. 10.1377/hlthaff.2014.0564 25006160

[24] Butler M McCreedy E Schwer N Burgess D Call K Przedworski J. Improving Cultural Competence to Reduce Health Disparities. AHRQ Comparative Effectiveness Reviews. Rockville, MD: Agency for Healthcare Research and Quality (2016). 27148614

[25] Jongen C McCalman J Bainbridge R. Health workforce cultural competency interventions: a systematic scoping review. BMC Health Serv Res. (2018) 18:232. 10.1186/s12913-018-3001-5 29609614 PMC5879833

[26] Govere L Govere EM. How effective is cultural competence training of healthcare providers on improving patient satisfaction of minority groups? A systematic review of literature. Worldviews Evid Based Nurs. (2016) 13:402–10. 10.1111/wvn.12176 27779817

[27] Davey MP Waite R Nunez A Nino A Kissil K. A snapshot of patients' perceptions of oncology providers' cultural competence. J Cancer Educ. (2014) 29:657–64. 10.1007/s13187-014-0619-9 24504662

[28] Committee Committee on Improving the Quality of Cancer Care: Addressing the Challenges of an Aging Population; Board on Health Care Services; Institute of Medicine Levit L Balogh E Nass S Ganz PA editors. Delivering High-Quality Cancer Care: Charting a New Course for a System in Crisis. Washington, DC: National Academies Press (2013). 24872984

[29] Ganz PA Hassett MJ Miller DC. Challenges and opportunities in delivering high-quality cancer care: a 2016 update. Am Soc Clin Oncol Educ Book. (2016) 35:e294–300. 10.1200/EDBK_159303 27249735

[30] Bonvicini KA. LGBT healthcare disparities: what progress have we made? Pat Educ Counsel. (2017) 100:2357–61. 10.1016/j.pec.2017.06.003 28623053

[31] McGarry KA Clarke JG Cyr MG Landau C. Evaluating a lesbian and gay health care curriculum. Teach Learn Med. (2002) 14:244–8. 10.1207/S15328015TLM1404_8 12395487

[32] Kelley L Chou CL Dibble SL Robertson PA. A critical intervention in lesbian, gay, bisexual, and transgender health: knowledge and attitude outcomes among second-year medical students. Teach Learn Med. (2008) 20:248–53. 10.1080/10401330802199567 18615300

[33] Shrader A Casero K Casper B Kelley M Lewis L Calohan J. Military lesbian, gay, bisexual, and transgender (LGBT) awareness training for health care providers within the military health system [formula: see text]. J Am Psychiatr Nurs Assoc. (2017) 23:385–92. 10.1177/1078390317711768 28569121

[34] Taylor AK Condry H Cahill D. Implementation of teaching on LGBT health care. Clin Teach. (2018) 15:141–4. 10.1111/tct.12647 28401669

[35] Wilsey CN Cramer RJ Macchia JM Golom FD. Describing the nature and correlates of health service providers' competency working with sexual and gender minority patients: a systematic review. Health Promot Pract. (2021) 22:475–90. 10.1177/1524839920943212 32698700

[36] Damaskos P Amaya B Gordon R Walters CB. Intersectionality and the LGBT cancer patient. Semin Oncol Nurs. (2018) 34:30–6. 10.1016/j.soncn.2017.11.004 29325815 PMC7424551

[37] Bauer GR. Incorporating intersectionality theory into population health research methodology: challenges and the potential to advance health equity. Soc Sci Med. (2014) 110:10–7. 10.1016/j.socscimed.2014.03.022 24704889

[38] Badgett MVL Durso LE Scheenbaum A,. New Patterns of Poverty in the Lesbian, Gay, Bisexual Community. (2013). Available online at: https://williamsinstitute.law.ucla.edu/wp-content/uploads/LGB-Poverty-Update-Jun-2013.pdf.

[39] Movement Advancement Project (MAP),. Paying an Unfair Price: The Financial Penalty for LGBT People of Color in America. (2015). Available online at: http://www.lgbtmap.org/file/payingan-unfair-price-lgbt-people-of-color.pdf.

[40] Lacombe-Duncan A. An intersectional perspective on access to HIV-related healthcare for transgender women. Transgend Health. (2016) 1:137–41. 10.1089/trgh.2016.0018 29159304 PMC5685282

[41] Seay J Hicks A Markham MJ Schlumbrecht M Bowman M Woodard J. Developing a web-based LGBT cultural competency training for oncologists: the COLORS training. Pat Educ Counsel. (2019) 102:984–9. 10.1016/j.pec.2019.01.006 30642714

[42] Hudson KD. Identity-conscious services for diverse patients: a descriptive analysis of lesbian, gay, bisexual, and transgender-focused federally qualified community health centers. J Gay Lesbian Soc Serv. (2018) 30:282–98. 10.1080/10538720.2018.1478353

[43] Cahill S Singal R Grasso C King D Mayer K Baker K. Do ask, do tell: high levels of acceptability by patients of routine collection of sexual orientation and gender identity data in four diverse American community health centers. PLoS ONE. (2014) 9:e107104. 10.1371/journal.pone.0107104 25198577 PMC4157837

[44] Grasso C McDowell MJ Goldhammer H Keuroghlian AS. Planning and implementing sexual orientation and gender identity data collection in electronic health records. J Am Med Inform Assoc. (2019) 26:66–70. 10.1093/jamia/ocy137 30445621 PMC6657380

[45] Ard KL Keuroghlian AS. Training in sexual and gender minority health - expanding education to reach all clinicians. N Engl J Med. (2018) 379:2388–91. 10.1056/NEJMp1810522 30575482

[46] Cahill S Makadon H. Sexual orientation and gender identity data collection in clinical settings and in electronic health records: a key to ending LGBT health disparities. LGBT Health. (2014) 1:34–41. 10.1089/lgbt.2013.0001 26789508

[47] Cahill SR Baker K Deutsch MB Keatley J Makadon HJ. Inclusion of sexual orientation and gender identity in stage 3 meaningful use guidelines: a huge step forward for LGBT health. LGBT Health. (2016) 3:100–2. 10.1089/lgbt.2015.0136 26698386

[48] Stirman SW Baumann AA Miller CJ. The FRAME: an expanded framework for reporting adaptations and modifications to evidence-based interventions. Implement Sci. (2019) 14:58. 10.1186/s13012-019-0898-y 31171014 PMC6554895

[49] Kamen C Smith-Stoner M Heckler CE Flannery M Margolies L. Social support, self-rated health, and lesbian, gay, bisexual, and transgender identity disclosure to cancer care providers. Oncol Nurs Forum. (2015) 42:44–51. 10.1188/15.ONF.44-51 25542320 PMC4360905

[50] Kamen C Alpert A Murray N Janelsins M Mohile S Mustian K. “My Partner is My Family”: Psychosocial Oncology Needs of Sexual and Gender Minority Cancer Patients and Their Caregivers. Society of Behavioral Medicine, Washington, DC (2019).

[51] Alpert AB Gampa V Lytle MC Manzano C Ruddick R Poteat T. I'm not putting on that floral gown: enforcement and resistance of gender expectations for transgender people with cancer. Pat Educ Counsel. (2021) 104:2552–8. 10.1016/j.pec.2021.03.007 33745786 PMC9320277

[52] SocioCultural Research Consultants LLC. Dedoose Version 9.0.17, Web Application for Managing, Analyzing, and Presenting Qualitative and Mixed Method Research Data. Los Angeles, CA: SocioCultural Research Consultants LLC (2021).

[53] Miller CJ Barnett ML Baumann AA Gutner CA Wiltsey-Stirman S. The FRAME-IS: a framework for documenting modifications to implementation strategies in healthcare. Implement Sci. (2021) 16:36. 10.1186/s13012-021-01105-3 33827716 PMC8024675

[54] Proctor EK Powell BJ McMillen JC. Implementation strategies: recommendations for specifying and reporting. Implement Sci. (2013) 8:139. 10.1186/1748-5908-8-139 24289295 PMC3882890

